# Penile strangulation injury by metallic ring: A study of 4 cases

**DOI:** 10.1016/j.ijscr.2021.01.103

**Published:** 2021-02-01

**Authors:** Bambang Sasongko Noegroho, Safendra Siregar, Rizky Ramdhani, Bernard Partogu, Akhmad Mustafa

**Affiliations:** Department of Urology, Faculty of Medicine Universitas Padjadjaran, Hasan Sadikin Hospital, Indonesia

**Keywords:** Penile strangulation, Metallic ring, Paraphilia

## Abstract

Penile strangulation by metallic ring is an emergency situation which requires urgent management. Strangulation of the penis usually performed in order to increase sexual performance or because underlying mental disorder or paraphilia. It is an uncommon urological emergency, if not treated as soon as possible it can lead to complications such as gangrene and amputation of the penis. There is no standard of care that has been found to be superior, with each case managed individually according to its clinical findings and operative settings. In this study, we present four cases of penile strangulation in last two years in a tertiary care hospital.

## Highlights

• Penila strangulation required emergency management to preserve the organ function.
• Each case managed individually according to its clinical finding and operative settings.
• Management depends on the type and size of constricting object, time after incarceration, degree of injury, available instrument, and experience of the physicians.
• Different methods and tools may arise due to circumstance and individual cases differences.
• Surprisingly, there is no erection problem after removing the strangulation.

## 1 Introduction

Penile strangulation by a foreign body is a rare condition [1]. Strangulation of the penis is usually associated with an attempt to improve sexual act by maintaining a prolonged erection. It is requires urgent intervention and treatment since it may affect vascular injury or even necrosis [2]. The objects which are usually used by adults and adolescents for penile entrapments are metallic and nonmetallic. Nonmetallic, thin objects can easily be cut off, but penile entrapment with heavy metal objects can pose difficult problem, especially as the object cannot be removed by the standard equipment in hospital [3].

There is no standard of care that has been found to be superior, with each case managed individually according to its clinical findings and operative settings [4]. Here we present a case series of four cases of penile strangulation that involve different onset, clinical presentation, surgical approach, and follow up. We collected the data retrospective from single centre. All surgical procedur was done by senior urologic resident. This article was made according to Preferred Reporting Of Case Series in Surgery (PROCESS) 2020 [5]. All participants already give written informed consent for the surgical procedures and the write up of the reports and use of images for publication.

## 2 Case presentation

### 2.1 Case 1

**Fig. 1:**
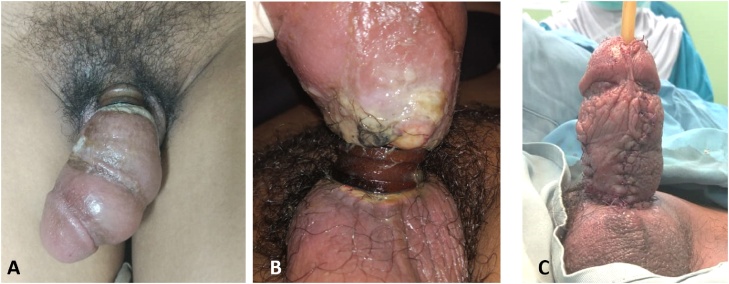
**A**. Penile strangulation by metal ring **B**. Skin ulceration because of chronic strangulation **C**. Ulceration of penile skin was repaired with necrotomy, debridement, and primary suture.

A 40 years old man came to Emergency Room with strangulated penile by a metal ring since 1 month ago. Physical examination showed the shaft of penis, which was distal to the ring, was edematous, congested, and purulent. The glans and distal shaft were reported to be viable with a well-demarcated line of bottle constriction. A wire plier was used to cut the ring. A gauze was used to cover skin. Following removal, the penile edema began to resolve and laceration of penile skin was repaired with necrotomy, debridement, and primary suture. Patient was followed-up on day 30 after procedure, voiding function was normal and Erection Hard Score (EHS) was 4 (Fig. 1).

### 2.2 Case 2

A 25 years old man came to Emergency Room with strangulated penile by a metal ring since 18 h ago. He complained unable to urinate and severe pain. Physical examination showed the shaft of penis, which was distal to the ring, was edematous and congested. The glans and distal shaft were reported to be viable.

**Fig. 2:**
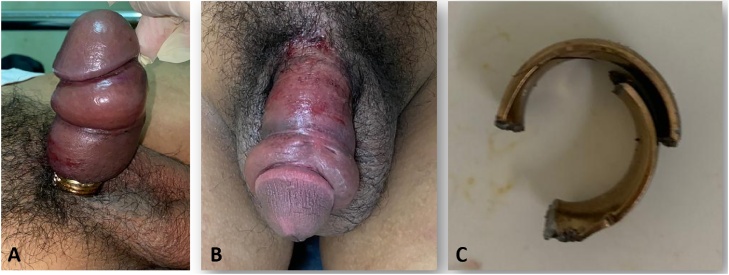
**A**. Penile strangulation; **B**. Post release strangulation; **C**. Metallic ring.

A pliers was used to cut the ring. A gauze was used to cover skin below to prevent more laceration. Following removal, the penile edema began to resolve and no signs of necrosis or damage to the penis were noted. Patient was followed-up on day 30 after procedure, voiding function was normal and Erection Hard Score (EHS) was 4 (Fig. 2).

### 2.3 Case 3

A 38 years old man came to Emergency Room with strangulated penile by a metal ring since 16 h ago. He complained unable to urinate and severe pain. Physical examination showed a strangulated penile due to a metallic ring at the base of his penis. The shaft of penis, which was distal to the ring, was edematous, congested. Its consistency was hard, had a dark colored accompanied by decreased penile sensation. The glans and distal shaft were reported to be viable with a well-demarcated line of bottle constriction. He had history of psycothic disorder.

**Fig. 3:**
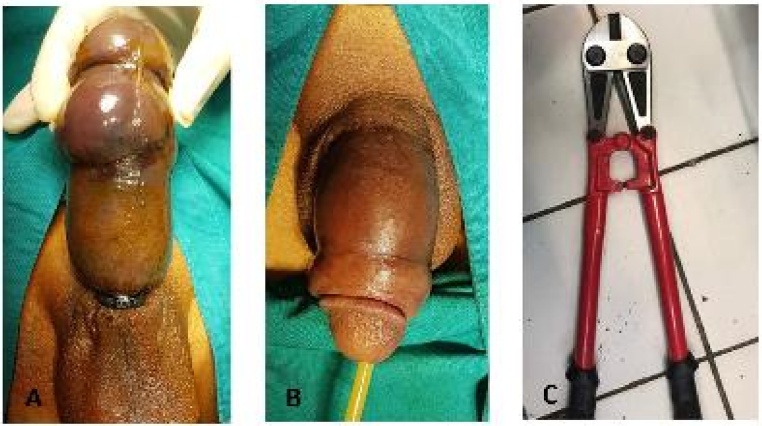
**A**. Penile strangulation by metalic ring; **B**. The penile edema began to resolve; **C**. Plier used to cut the ring.

A pliers was used to cut the ring. Before the procedure, a gauze was used to cover skin below to prevent more laceration. Following removal, the penile edema began to resolve and no signs of necrosis or damage to the penis were noted. Patient was followed-up on day 30 after procedure, voiding function was normal and Erection Hard Score (EHS) was 4 (Fig. 3).

### 2.4 Case 4

A 26 years old man came to Emergency Room with strangulated penile by a metal ring since 1 month ago. He complained severe pain and swelling at penis. Physical examination showed a strangulated penile due to a thick metallic ring at the base of his penis. The shaft of penis, which was distal to the ring, was edematous and congested. The glans and distal shaft were reported to be viable with a well-demarcated line of bottle constriction.

**Fig. 4:**
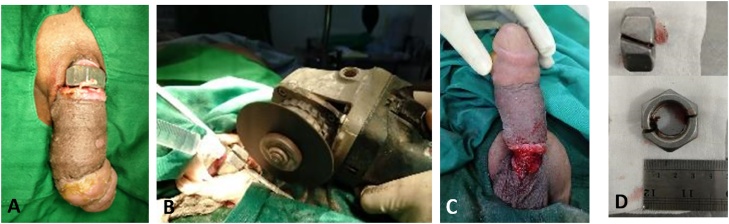
**A**. Strangulated penis by metallic ring; **B**. Grinder, used to cut the ring; **C**. Lacerated skin due to chronic strangulation; **D**. Thick metallic ring.

This is the biggest metal ring that we found in our cases with the diameter of the ring is 40 mm and the thickness of the ring is 5 mm. We have made a section of the metallic ring by a grinder. A metal tongue depressor was inserted between the penis and the ring. Continuous irigation during removal with cold sterile water was done to prevent thermal injury. Following removal, the penile edema began to resolve and laceration of penile skin was repaired with primary suture. Patient was followed-up on day 30 after procedure, voiding function was normal and Erection Hard Score (EHS) was 3 (Fig. 4).

## 3 Discussion

Penile strangulation injuries have been reported worldwide [2]. In middle-aged and elderly men, the leading reason of application these foreign bodies are to increase sexual performance, increase sexual arousal, or because of autoerotic intentions.^3^ Gauthier reported the first case of penile strangulation in literature in 1755 [5]. Since then, approximately 60 cases have been reported in the world literature [2].

Strangulated penile is an emergency situation that may lead to a wide range of vascular and mechanical injuries [6]. Urgent treatment is required, as potential delayed management may lead to mild, reversible vascular obstruction, lymphedema, loss of penile sensation, ischemic, skin necrosis/ulceration, urethrocutaneous fistula, urethral injury, gangrene, and even autoamputation of penis and sepsis [6]. After that, mainly the treatment aimed to decompressing and restoration of the penile circulation [7]. In this case, there were engorged penile shaft distal to the ring, diminished of sensation and 2 cases accompanied with skin ulceration.

As the constricting devices involved are variable, physician have to more creative due to one technic are neither applicable nor available in each case. Many methods have been reported for removal penile foreign body [6,8]. First, engorged penile make the foreign body cannot slip off easily, so physician have to put a string (or any other object) to make a little space between constricting devices and skin. In this case, we used a gauze to protect the skin and also to made it slip off easier. Second, constricting devices may be too hard due to be severed by common surgical instruments. Due to its condition, the tools needed for removal of constricting devices may not universally available in emergency room. Since it was located completely on base of penis, it was more dangerous if we used a hacksaw and also too hard if we used string technique only. Subsequently, we used a pliers to cut off the ring. After the ring removal, penile was directly flaccid.

In 1991, Bhat et al. presented a classification for penile incarceration composed of five grades as follows [7,9].

• Grade I: Edema of the distal penis.
• Grade II: Injury to the penile skin constriction of corpus spongiosum without any urethral injury. Distal penile edema with decrease sensation.
• Grade III: Injury to skin and urethra but no urethral fistula. Loss of distal penile sensation.
• Grade IV: Complete division of corpus spongiosum leading to urethral fistula and constriction of corpus cavernosa with loss of distal penile sensation.
• Grade V: Gangrene, necrosis, or complete amputation of the distal penis.

Based on this classification, 2 patient was in grade I and 2 patient was in grade II. Voiding function was also back to normal after 2 h of releasing (Table 1).

**Table 1:** Characteristic of the patient.

Patient Number	Age	Motive	Applied by	Object	Urinary retention	Grade of injury	Duration	Management	EHS	Mental disorder	Complication
1	40	Sexual gratification	Self	Metalic ring	–	2	1 Month	Wire plier and primary suture	4	Schizophrenia	–
2	25	Sexual gratification	Self	Metalic ring	Yes	1	18 h	Wire plier	4	–	–
3	38	Sexual gratification	Self	Metalic ring	Yes	1	16 h	Wire plier	4	Schizophrenia	–
4	26	Sexual gratification	Self	Metalic ring	–	2	1 Month	Grinder and primary suture	3	–	–

Depending on the degree and material of entrapment and distal edema caused by it, releasing it can be challenging. If the constricting object is nonmetallic object, it can be easily cut off, but thick, hardened steel or iron is very difficult to remove with a chisel, saw, or cutter [6].

Cutting metal produces heat, and to prevent tissue heating, the metal must be cooled. The penis itself must be protected during cutting, which can be difficult because there is usually little room between the metal and penis. Likewise, the metal must be cut in two spots to avoid damage to the penile skin during removal [10]. In all our case, we continuously sprinkled cold normal saline to cool both the penile tissue. In last case we use an angel grinder to remove the metallic ring. We inserted a metal tongue-shaped blade between the strangulating ring and penile skin which prevented from penile skin and tissue injury from the heat and force. It cuts very smoothly in a short duration without much exertion as it is operated electrically.

## 4 Conclusion

Penile strangulation required emergency management to preserve the organ function. Each case managed individually according to its clinical findings and operative settings. Management depends on the type and size of the constricting object, time after incarceration, degree of injury, available instruments, and experience of the physicians. Different methods and tools may arise due to circumstance and individual cases differences. Suprisingly, there is no erection problem after removing the strangulation.

## Declaration of Competing Interest

The authors report no declarations of interest.

## Funding

This research received no specific grant from any funding agency in the public, commercial, or not-for-profit sectors.

## Ethical approval

There is no ethical clearance needed for this manuscript according to local hospital ethic committee.

## Consent

• Permission has been obtained from the copyright holder to reproduce in the work (in all media, including print and electronic form) material not owned by the authors, and the source has been acknowledged.
• This manuscript contains no violation of any existing copyright or other third-party right or any material of an obscene, indecent, libelous, or otherwise unlawful nature, and to the best of the authors’ knowledge, this work does not infringe the rights of others.

## Author contribution

BSN: study concept or design, writing the paper.

SR: study concept or design.

RR: data collection, writing the paper.

## Registration of research studies

Not applicable.

## Guarantor

This study is guaranteed by Bambang S Noegroho, MD, PhD.

## Provenance and peer review

Not commissioned, externally peer reviewed.
